# Developing Critical Appraisal and Evidence Synthesis skills in future Microbiologists

**DOI:** 10.1093/femsle/fnab114

**Published:** 2021-10-07

**Authors:** Matthew Flynn

**Affiliations:** Department of Otolaryngology, Head and Neck Surgery, NHS Greater Glasgow and Clyde; University of Edinburgh Medical School

**Keywords:** Microbiology, Education, Evidence-Based Medicine, Review Literature, Research Activities

## Abstract

The Covid-19 pandemic has demanded modifications to undergraduates’ learning experiences, and promised a more challenging scientific world in which they will live. Bespoke evidence synthesis and critical appraisal skills modules are an opportunity to utilise our information-saturated world to our advantage. This programme of study made use of a virtual journal club, structured literature searches, scoping review methods and a variety of online research tools to navigate and critique the literature. The program design is here outlined with sample learning objectives and reference to the resources used.

ORIGINAL UNEDITED MANUSCRIPT

*Main Text*

Medical and biomedical science education have a very pressing problem: aside from the ever broader and deeper knowledge base to be mastered by todays' undergraduate, the problems which these students will face as graduates are increasingly difficult, surrounded by a mounting body of literature of uncertain quality. The Covid-19 Pandemic has been a prime example of this global uncertainty and abundance of conflicting evidence. Future citizens will have to be more comfortable with risk and uncertainty, whilst being literate in assimilating and sifting scientific literature for themselves[1,2]. 'Wicked' problems, such as antimicrobial resistance, are defined by having no known solving-point, being worldwide, unable to be solved by trial-and-error, evolving, hard to define and containing symptoms of co-related problems[3]. The next generation must be equipped to tackle these by moving beyond 'factual literacy' to 'critical science literacy,' able to both reconcile the competing findings of their day and interpret them for a lay audience[4]. Todays' undergraduate is already significantly more adept than the preceding generation at sifting through large quantities of information and potentially contrarian views through their use of social media: the largest cohort of twitter users is in the 18-29 age bracket[5].

Higher education courses are increasingly producing graduates with transferrable skills and published work. Classroom undergraduate research experiences (CURES) offer engagement with open ended questions and increase student engagement, with many choosing to become involved beyond the end of the project[6,7]. After one such gut microbiome CURE embedded within an undergraduate microbiology course, 88% of the students stated that the material that they learned was useful for their future careers[8]. Medical education is particularly burdened with developing critical appraisal skills in a stepwise manner throughout the degree course, as a prerequisite to being able to select effective treatments based on the best available evidence[9,10]. Here are addressed the skills of critical appraisal and basic evidence synthesis and how they were developed within short student microbiologically focussed modules in an undergraduate medical course.

Blooms' taxonomy places "analysis" and "evaluation" toward the apex of Educations' objectives, building on a foundation of understanding and application[11]. A way to develop these is through critical appraisal student selected component (SSC) modules. This was run by the university department alongside the regular 2nd year medicine curriculum, with an over-arching topic chosen by the tutors, in my case "Potentially protective bacteria of the upper respiratory tract." The weekly online meetings followed a 'virtual journal club' format. Each week a different student presented a different article which was then discussed with the group; these spanned from seminal papers and review articles through to very recent developments. Over the course of 10 1-hour sessions, a body of literature was discussed, with a nominated facilitator leading a critique of researchers' methods and the validity of their conclusions. The Critical Appraisal Skills Programme (CASP) checklists are a very useful tool to decipher articles in this regard[12]. Different students then completed a paragraph on whichever aspect most interested them, together compiling a mini-review which was submitted for the final assessment. The weekly discussions allowed time for the tutor to give feedback on their students' analytical and appraisal skills as formative assessment. 4 of the 9 students expressed an interest in doing further study or publication in this area following the completion of the module. A similar virtual journal club model has been used, where participants contribute asynchronously to a critique which may be submitted as a Letter to the Editor[13]. Learning outcomes for the block included:

1. Learn the different types of literature and study design
2. Critically appraise a paper against a quality checklist
3. Contribute to group discussions on papers presented by other students
4. Develop an area of interest within upper respiratory tract microbiology
5. Summarise some of the key findings within this niche, with comments on any major risk of bias or applicability concerns of the contained articles.

Evidence Synthesis

Evidence synthesis is the aggregation of relevant scientific literature as it pertains to a precise question. This is a key skill for clinicians whose actions and decisions are based on information. The classic systematic review (the 'whole truth and nothing but the truth' on a topic) has been supplemented more recently by scoping reviews and rapid reviews. Scoping reviews search the literature broadly to quantitively summarise the number of papers on a subject over time, and qualitatively outline key themes and gaps within it. Scoping reviews are particularly apt for undergraduates, as quick to perform, and introducing key skills such as defining a research question, literature searching and making inclusion/exclusion criteria decisions[14]. The question "Which are the most common presenting signs and symptoms for Epiglottitis" was answered by doing a structured literature search of the above terms and comparing the frequency of the 18 most mentioned clinical indicators of this condition within the resultant articles. Such overviews can expose areas requiring further study, and identify promising combinations of symptoms for scoring systems like the recently constructed Liverpool Peritonsillar Abscess Score[15]. This particular project was appropriate learning for a 1st year cohort, as it gave an overview of this condition in its most basic terms. The students surveyed 234 full text articles from Medline and Cochrane in March 2020 and summarised the most frequently mentioned signs and symptoms of epiglottitis [fig. 1].

Learning objectives for the block included:

1. Asking a simple research question
2. Deciding the scope of the research (inclusion criteria)
3. Establishing the closely related/overlapping topics not of interest (exclusion criteria)
4. Deciding how comprehensive to make the analysis (e.g. whether to include grey literature, how many years of publication)
5. Using scientific databases and evidence synthesis software to collate results.

Evidence synthesis has been a valuable source of research projects for students over the Covid-19 pandemic where 'wet-lab' and near-patient opportunities have been in short supply. Freely available web apps such as Rayyan QCRI allow students to collaborate asynchronously on a portfolio of articles by excluding papers, assigning labels and applying filters[16].

Outlook

Undergraduate critical appraisal and evidence synthesis projects can be powerful tools in student engagement. Scientific literature becomes less daunting as students learn to navigate it within a community of practice. Such projects help students identify both methodological strength and potential gaps in the literature: fertile grounds for future clinician-scientists. Their presence as an accompanying thread throughout undergraduate degrees rather than as a temporary project will help engagement with literature become a part of everyday practice[17]. The Covid-19 pandemic has provided a unique stimulus for these types of CURES to prove their efficacy and help produce future-ready graduates.

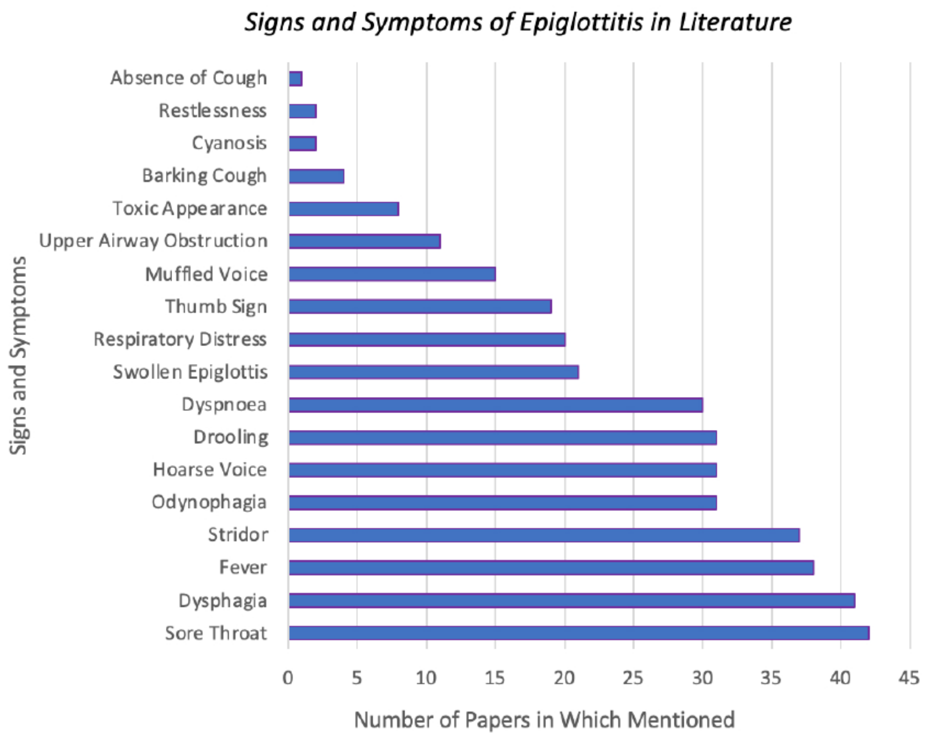

Figure 1
The seven most referenced signs and symptoms of epiglottitis in 234 relevant full text articles from Cochrane and Medline.

ORIGINAL UNEDITED

*Funding*
No sources to Declare

*Conflict of Interest*
None to Declare

*Acknowledgements*
The year 2 MBChB course at Edinburgh has been designed by Prof. J. Plevris and Prof S Riley, and lead by Dr S. Mallen.

ORIGINAL UNEDITED MANUSCRIPT
